# Drone exploration of bat echolocation: A UAV‐borne multimicrophone array to study bat echolocation

**DOI:** 10.1002/ece3.9577

**Published:** 2022-12-03

**Authors:** Christian Jespersen, David Docherty, John Hallam, Carsten Albertsen, Lasse Jakobsen

**Affiliations:** ^1^ Department of Biology University of Southern Denmark Odense M Denmark; ^2^ Maersk McKinney Moller Institute University of Southern Denmark Odense M Denmark

**Keywords:** bats, echolocation, microphone array, source level, UAV

## Abstract

Multimicrophone array techniques offer crucial insight into bat echolocation, yet they severely undersample the environments bats operate in as they are limited in geographic placement and mobility. UAVs are excellent candidates to greatly increase the environments in which such arrays can be deployed, but the impact of UAV noise on recording quality and the UAV's behavioral impact on the bats may affect usability. We developed a UAV‐borne multimicrophone setup capable of recording bat echolocation across diverse environments. We quantify and mitigate the impact of UAV noise on the recording setup and test the recording capability of the array by recording four common Danish bat species: *Pipistrellus pygmaeus*, *Myotis daubentonii*, *Eptesicus serotinus*, and *Nyctalus noctula*. The UAV produces substantial noise at ultrasonic frequencies relevant to many bat species. However, suspending the array 30 m below the UAV attenuates the noise to levels below the self‐noise of our recording system at 20 kHz and above, and we successfully record and acoustically localize all four bat species. The behavioral impact of the UAV is minimal as all four species approached the array to within 1 m and all emitted recordable feeding buzzes. UAV‐borne multimicrophone arrays will allow us to quantify bat echolocation in hitherto unexplored habitats and provide crucial insight into how bats operate their sonar across their entire natural habitat.

## Graphical abstract

We developed a UAV‐borne multimicrophone setup capable of recording bat echolocation across diverse environments. The system will allow us to quantify bat echolocation in hitherto unexplored habitats.

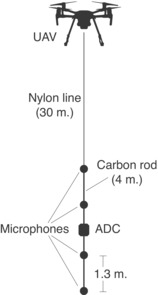

## 1 INTRODUCTION

All animals are inherently adapted to their specific habitat, and to understand their behavior fully, it is important to study them in their natural habitat. However, complex experimental design favors highly controlled laboratory conditions and detailed knowledge is often restricted to such laboratory settings. This is also the case for bat echolocation. Echolocation calls are clearly adapted to habitat and adjusted to suit changing environments and behavioral context (Schnitzler & Kalko, 2001). Bats flying in the open emit calls of substantially higher source level, longer duration, lower frequency, and lower call rate compared to flying in close quarters (Hulgard et al., 2016; Jensen & Miller, 1999; Surlykke & Moss, 2000) and bats approaching objects or prey will rapidly adjust their calls, reducing source level and duration, and increasing call bandwidth and call rate up to 200 calls/s and above in the terminal buzz immediately before prey capture (Griffin et al., 1960). Yet, most in‐depth knowledge of bat echolocation comes from laboratory experiments where the echolocation call repertoire is limited to calls suitable for very confined spaces (Hulgard et al., 2016; Surlykke & Moss, 2000). It is therefore clear that many aspects of echolocation such as species‐specific echolocation adaptations to habitat cannot be investigated in the laboratory.

Recent years have seen an increase in field recordings with the development of microphone array techniques, and our knowledge of the natural repertoire of echolocating bats is steadily growing (Hulgard et al., 2016; Jensen & Miller, 1999; Koblitz, 2018; Seibert et al., 2015; Surlykke & Kalko, 2008). Array recordings hold several advantages over traditional single‐microphone survey setups. The major advantage is the ability to localize the calling bat (Koblitz, 2018). Single microphone recordings do yield spatial information but, without the knowledge of location, the bat can be in any direction around the microphone out to the maximum detection distance dictated by the microphone's sensitivity and directionality and by the source parameters of the bat (source level, frequency, and directionality) (Ratcliffe & Jakobsen, 2018). The array provides an accurate estimate of the bat's location at the time of call emission and thus allows us to relate all source parameters to where the bat is in space and how it is moving. The location of the bat then allows us to compute source levels and with enough receivers also the direction and shape of the echolocation beam (Jakobsen et al., 2015; Jakobsen & Surlykke, 2010; Koblitz, 2018; Kounitsky et al., 2015; Seibert et al., 2015) which provides crucial insight into how the bat senses its environment through echolocation.

However, even with recent methodological advances, it is very apparent that array techniques severely undersample the habitats used by bats. Standard array setups are limited in their geographic placement, that is, near the ground or mounted on trees and other structures, and they are, unlike the bats, not movable within a short time period and thus cannot actively adapt to the bats' changing movement patterns. Several studies show that many bats fly above the canopy and some species fly up to, and above, 1000 m with peak activity around 500 m above ground. There are even reports of bats flying up to 3000 m above ground (Gillam et al., 2009; Griffin & Thompson, 1982; McCracken et al., 2008; Williams et al., 1973). Array techniques are generally limited to the localization of echolocating bats within ~30 m of the array (Holderied & von Helversen, 2003; Hulgard et al., 2016; Koblitz, 2018; Surlykke & Kalko, 2008).

The development of miniaturized bat‐borne acoustic tags rectifies many of the limitations possessed by large microphone arrays as data are recorded from any habitat the bat moves in while wearing the tag (Cvikel et al., 2015; Stidsholt et al., 2019; Stidsholt, Greif, et al., 2021; Stidsholt, Johnson, et al., 2021). But bat‐borne acoustic tags are also limited by recording off axis and by the carrying capacity of bats, currently limiting the use of tags to bats above 12 g at best (Egert‐Berg et al., 2018; Stidsholt et al., 2019). This restricts their general usability given that the median estimated body mass for all bats (including the large non‐echolocating pteropodids) is 12 g and the mode is 5 g (Arevalo et al., 2020).

Unmanned aerial vehicles (UAVs) are used increasingly in ecological surveys as they offer great speed, maneuverability, and precision, and they can easily explore environments not readily available to traditional survey methods (Fu et al., 2018; Scholten et al., 2019; Svane, Flindt, et al., 2022; Svane, Lange, et al., 2022; Wilson et al., 2017). As such, combining ultrasonic recordings with UAVs presents an opportunity to overcome the major limitations of traditional microphone array recordings, that is, they are stationary and ground based. There are, however, two major concerns associated with using UAVs for acoustic recordings: (i) The noise produced by the UAV may severely limit their usability by decreasing the sensitivity of the recording system because of masking noise (i.e., raising the noise floor). (ii) The presence of the UAV may deter bats from the area either because of masking noise or by the presence of a large flying object. Several studies have investigated the potential of UAVs as bat survey tools (i.e., single microphone presence/absence data) and they have addressed these concerns with varying results. All studies use mitigation efforts to reduce noise by shielding the microphone (August & Moore, 2019; Ednie et al., 2021; Fu et al., 2018; Kloepper & Kinniry, 2018) and/or suspending the microphone at some distance from the UAV (Michez et al., 2021). Both solutions yield clear improvements, but proper noise quantification is lacking. Noise is generally measured on a relative scale with uncalibrated equipment and not with any noteworthy frequency distinction. Reports of the behavioral effect of the UAVs show a reduction in bat activity when UAVs are flown (Ednie et al., 2021; Kuhlmann et al., 2022), but that the impact can be reduced by using smaller drones (Kuhlmann et al., 2022). There are no reports of natural foraging behavior recorded with any of the UAV setups.

The aim of our study is to develop a UAV‐borne large‐scale microphone array and investigate whether it can adequately record and quantify bat echolocation calls, such that it can be deployed in environments where standard arrays cannot operate. In doing so, we aim to quantify and mitigate the UAV noise recorded by the array and investigate if bats will approach and forage close to the UAV‐borne array.

## 2 METHODS

### 2.1 Array design

The recording setup was constructed from a customized Raspberry Pi Compute Module (RPi), using an SD card for memory, a lithium ion battery as power supply, and two USB ports for a Wi‐Fi transmitter and pressure, humidity, and temperature sensor (MSR 145 W, MSR Electronics GmbH). Four ports on the board connect to Knowles FG‐236229‐36 microphones. Each microphone had 20 dB preamp gain and its own programable amplifier (AD8336) allowing for additional 60 dB amplification. Each microphone signal was filtered by a 151‐kHz second‐order anti‐aliasing filter and either a 16‐kHz first‐order or 9‐kHz second‐order high‐pass filter. The four microphone channels were then sent through a multiplexer (MAX4638ETE+ 8:1 low‐voltage multiplexer) which channeled all four signals into a single audio bus. The bus was digitized using an LTC2205 A/D converter mounted on the board. The custom board presented the same interface as a USB‐DUXFast (Incite Technology Ltd). Data were read from the hardware using the standard Linux Comedi ADC subsystem. To operate at the high data rate required by this application, our custom board used a modified Linux kernel driver module, which is available from the authors. The raw audio from the A/D converter was encoded on the RPi using the audio processing tool SoX (http://sox.sourceforge.net/). The final output of the RPi was a single four‐channel wav file. The A/D can sample at 2.7 MHz across all channels, that is, maximum of 675 kHz per channel. Data were retrieved either through the Wi‐Fi connection with the RPi or by direct connection with the SD card. The board was encased in a plastic box alongside its battery pack and silica gel bags to absorb moisture (see Table S1 for component list and Figure S1 for board schematic). The combined price of the recorder and microphones was approximately 1500 euro excluding manufacturing costs. It may not be practical or feasible to replicate the recorder exactly as specified given technological development and changes in component availability, but it should be possible to construct a similar or improved system within the same price range.

**FIGURE 1:**
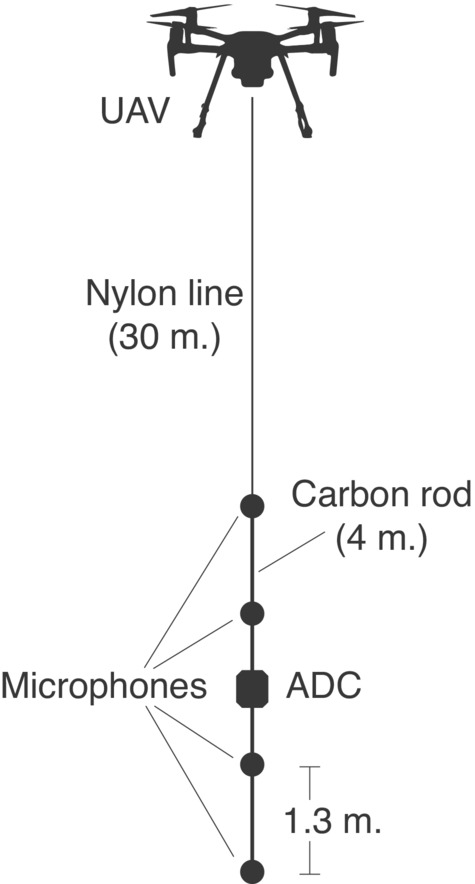
Schematic of the UAV and array setup.

The microphones and A/D converter were mounted on two linearly connected 2‐m carbon tubes (4‐m total length) with the A/D converter placed centrally and the microphones placed 1.30 m apart (Figure 1). The complete array had a total weight of 350 g—the two carbon tubes weigh 180 g and the microphones and recorder weigh 170 g.

### 2.2 UAV

We used a DJI M210 V2 UAV with a carrying capacity of 1340 g for our recordings, but any UAV with adequate carrying capacity should be usable. The DJI M210 V2, like many others, has a build‐in sonar that emits 40 kHz sound pulses which is problematic when recording bat echolocation as it overlaps with the call frequency of many species (Jones, 1999). Unfortunately, the UAV emits sound even when disabled via the controller and disabling it mechanically voids the warranty. We found that attaching a 40‐mm foam pad on the underside of the UAV attenuated the sound completely for our final recording setup.

### 2.3 Calibration

Before each recording session (noise test or field recording), we calibrated the microphones against a GRAS 40 BF ¼″ microphone using an Avisoft Scanspeak loudspeaker. We played a 10 ms, frequency‐modulated sweep (140 kHz –5 kHz) and computed the difference spectra between the GRAS and the Knowles microphones and subsequently converted the difference spectra into an impulse response which we used to filter the recordings.

### 2.4 Noise recordings

We performed noise recordings in an open field at the University of Southern Denmark, Odense, using a 1‐, 10‐, and 30‐m tether between the UAV and the top of the array (0.5 mm nylon fishing line, 18.4 kg carry capacity) with the foam shielding in place, and additionally without foam shielding below the drone using the 10‐m tether. For all noise recordings, we recorded 10‐s segments at 375 kHz sample rate. We additionally recorded the noise floor of the recording system by embedding the microphones in foam, preventing any sound from reaching the membrane.

### 2.5 Bat echolocation recordings

We recorded bats at four selected sites around Odense, Denmark, from June to September, 2021, two local parks, a field surrounded by trees where *Nyctalus noctula*, *Eptesicus serotinus*, and *Pipistrellus pygmaeus* are commonly found foraging, and a pond where *Myotis daubentonii* is commonly found foraging. We set up for recordings at sunset, and once bats were detected (by sight or a Petterson D200 ultrasound detector), the equipment was given the record command via Wi‐Fi and the UAV sent to the desired altitude. Each recording was 10 min long. Using three sets of DJI TB55 batteries for the UAV, we had a total flight time of ca. 60 min. Including battery swaps, the 60 min of flight time, usually resulted in ca. 90 min real time spent at the site which matched the bats active window quite well, that is, typically shortly after sunset and for around 90 min.

The linear construction of the array allows us to calculate altitude and distance to the bat relative to the array and consequently compute the source level of the bats in addition to the standard parameters afforded by single‐microphone recordings, that is, call duration, frequency content, and call interval. We calculated the location of the bats at each call emission using triangulation of the difference in arrival time of each call on the microphones (see e.g., Madsen & Wahlberg, 2007). We then compensated the recording on each microphone for transmission loss by filtering with the impulse response of the frequency‐dependent attenuation and multiplying with the spherical attenuation for each call. Afterward, we calculated the root mean square (RMS) sound pressure of each call on each microphone using the 95% energy content of an appropriately sized window containing each call; the size of the window varies with species and call type.

## 3 RESULTS

### 3.1 UAV noise

**FIGURE 2:**
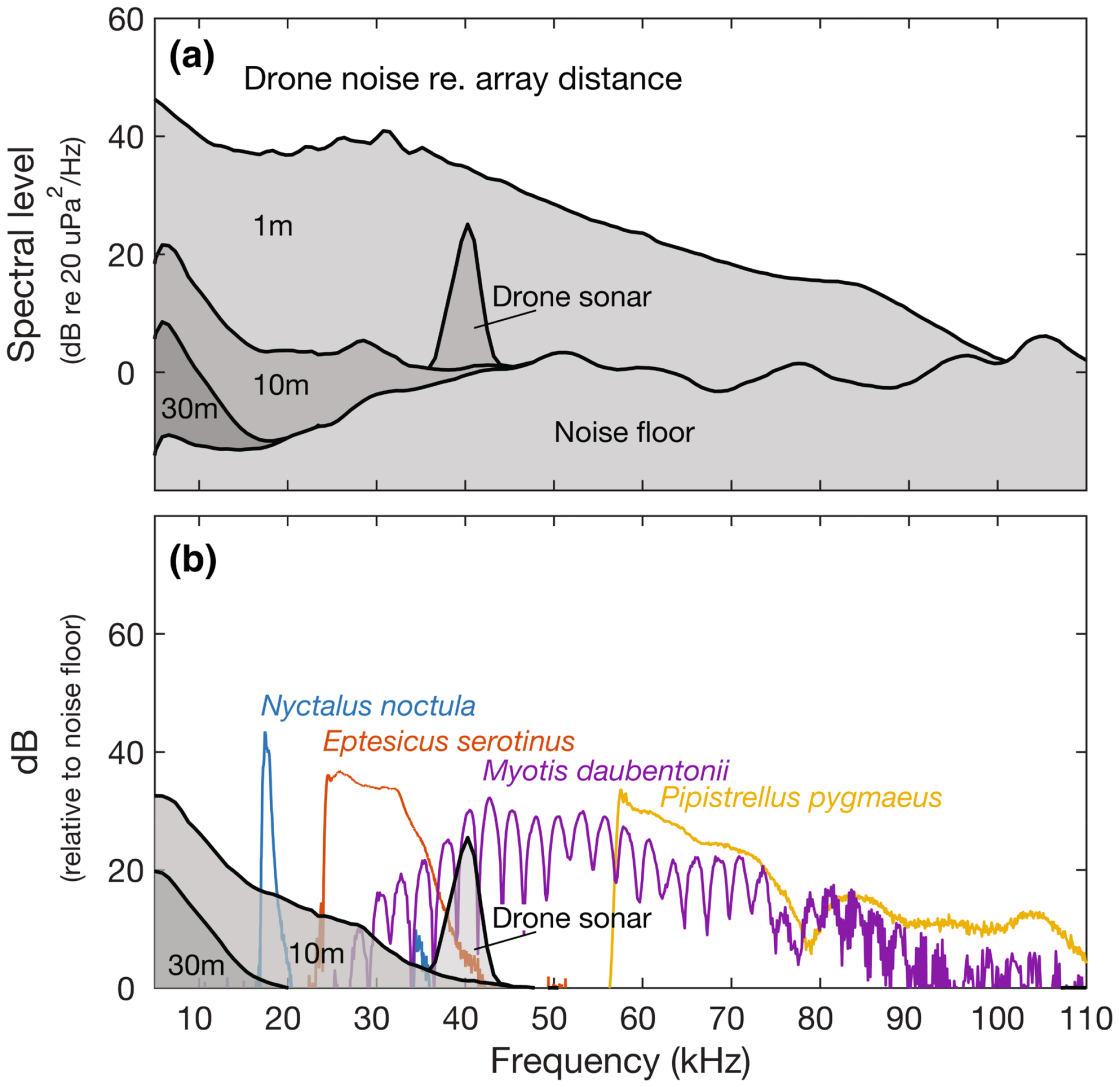
UAV noise profile and noise floor of the recording setup using a 512‐point running FFT with 512 sample window length and overlap of 256 samples. (a) Noise recorded at 1 and 30 m with padding attached and at 10 m with and without foam padding to attenuate the drone sonar. (b) Noise levels at 10 and 30 m relative to the noise floor of the recording system, with spectrum representation of typical search calls from the four species recorded.

The UAV clearly produced noise in the same spectrum as the bat echolocation calls. Even with the10‐m tether, drone noise in the region of the lowest frequency emitted by *N. noctula* (peak frequency around 18 kHz; Russ, 2021) was roughly 16 dB above the noise floor of the system, and for *E. serotinus* calls (peak frequency around 25 kHz; Russ, 2021), it was roughly 12 dB above the system noise floor (Figure 2). However, with the 30‐m tether, drone noise was below the system noise floor from 20 kHz and up, and at 18 kHz, it was just 0.5 dB above the noise floor of the system. The drone sonar was also efficiently attenuated below the noise floor by the foam padding even when using the 10‐m tether (Figure 2).

### 3.2 Bat recordings

**FIGURE 3:**
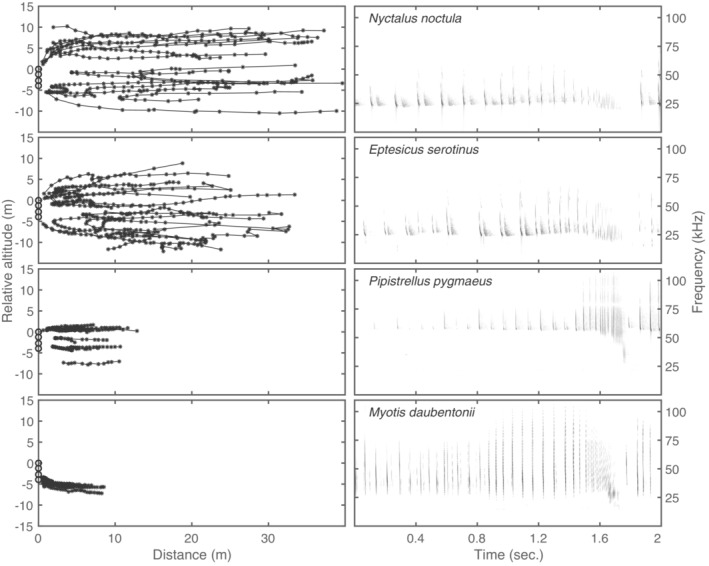
Flight paths and exemplary spectrograms for foraging attempts by all four recorded bat species. Each flightpath plot contains 10 flightpaths; calls are indicated by * and connected by lines within each flightpath; microphones are indicated with circles.

Based on our noise recordings, we used the 30‐m tether for all field recordings. We obtained recordings from four common Danish bat species at the four selected field sites: *N. noctula*, *E. serotinus*, *M. daubentonii*, and *P. pygmaeus*. All bats flew within 1 m of the microphone array, and we recorded feeding buzzes for all four species (Figure 3).

**FIGURE 4:**
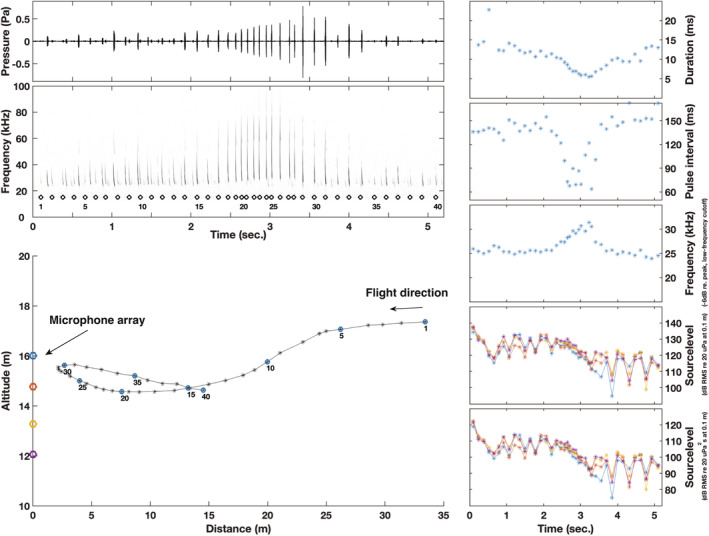
Exemplary recording of a serotine bat (*Eptesicus serotinus*) with localization and quantification of echolocation parameters. Source levels are color coded corresponding to the respective recoding microphone as indicated in the flightpath plot.

We computed source parameters for a selected flight path for *E. serotinus*, as it flew past the array (Figure 4). The recording shows standard search calls for this species, with call duration around 15 ms, pulse interval around 150 ms, and minimum frequency at around 25 kHz. At ca. 10 m from the array, the bat starts exhibiting approach behavior, reducing duration and pulse interval, and increasing call frequency. Emitted source levels likewise show changes corresponding to approach behavior: pressure values are around 130 dB (RMS re 20 μPa at 0.1 m) for search calls when the bat flies toward the array and pressure drops alongside changes to duration, pulse interval, and frequency within 10 m of the array. Energy logically follows changes to duration and pressure. The alternating source levels as the bat flies toward the array indicate scanning behavior, and given that the pattern is identical across all microphones, it indicates that the bat is scanning from side to side, not up and down. The scanning behavior is also apparent when the bat flies away from the array, but here source levels are clearly lower, likely because of call directionality (Figure 4).

## 4 DISCUSSION

Our recordings clearly show that UAVs are viable tools for array recordings of most bat species on par with conventional stationary array solutions. The simple four microphone setup we employed allows us to calculate source levels and rudimentary beam direction in addition to standard call parameters (duration, call rate, and frequency). Existing UAV systems with ultrasonic microphones only provide standard call parameters with very limited information about the bats location (within detection range of the drone). With our system, we can relate all standard parameters as well as source level and beam measurements to accurate estimates of the bats location in space, allowing us insight into how bats operate their sonar in environments previously unexplored by array systems. The simple linear setup with four microphones can easily be expanded upon. The DJI M210 V2 that we use can carry 1340 g which means that we can comfortably expand the array setup to encompass three such four microphone arrays. We have performed preliminary trials using a carbon frame that holds 12 microphones in a three by four microphone configuration as well as an extended linear configuration with eight and 12 microphones each consisting of the base 4‐m four microphone setups separated by 6‐m nylon string. All configurations were easily handled by the UAV albeit with a decrease in flight time corresponding to the increase in weight. Utilizing different array configurations will also allow us to localize the bats in 3D instead of 2D as is the case with the linear array, but the linear array benefits from fewer zones with poor localization accuracy than 3D capable configurations (Madsen & Wahlberg, 2007) and the 2D localization fulfills our current requirements, that is, distance and direction allow us to measure source level in relation to flight height above ground level.

We show that very simple noise mitigation allows us to record even low‐frequency species with little to no impact from UAV noise. We further show that the behavioral impact of the UAV is limited, given that all four species approach the microphone array within 1 m and all four species emit recordable feeding buzzes. Compared to previous reports, we use a much greater distance between the UAV and the microphones (August & Moore, 2019; Ednie et al., 2021; Fu et al., 2018; Kloepper & Kinniry, 2018; Michez et al., 2021), which reduces the noise substantially and very likely also the behavioral impact of the UAV considerably. Previous studies report avoidance behavior and a clear reduction in bat activity in the presence of UAVs with no reports of the recorded foraging that we observe for all recorded species (Ednie et al., 2021; Fu et al., 2018; Kloepper & Kinniry, 2018; Kuhlmann et al., 2022). Furthermore, we are relatively stationary during the actual recording, that is, we move the UAV to the desired location and remain there for the duration of the recording, and we return to this spot throughout the whole recording night with minor modifications to the specific altitude. This recording method may facilitate habituation by the bats to a much greater degree than the recording methods described in previous studies, that is, moving UAV with the microphone directly attached or suspended a short distance away (Ednie et al., 2021; Fu et al., 2018; Kloepper & Kinniry, 2018; Kuhlmann et al., 2022). While this strategy works very well for our purposes, it may not be applicable to surveys if the purpose is to investigate bat activity in as large an area as possible and other mitigation measures may be more appropriate. Such mitigation measures could include modifications to the motors and rotor blades to reduce high‐frequency noise, or even a switch away from quad‐copter UAVs to fixed wing UAVs or blimps.

Our preliminary results for *E. serotinus* show comparable measures for call rate, frequency content, and duration to previous reports at the same altitude (Jensen & Miller, 1999) and the emitted source levels are also comparable to previous measures (Holderied & von Helversen, 2003), although differences in SL computation (peak equivalent RMS by Holderied & von Helversen, 2003 vs. our RMS) indicate that we measure slightly higher output levels. This also makes sense given that we measure at the same altitude as the bats instead of from the ground, that is, the directional emission of the bats make it more likely to measure off‐axis at ground level (Jakobsen, Brinklov, et al., 2013; Jakobsen, Ratcliffe, et al., 2013; Surlykke et al., 2009). The inferred side‐to‐side scanning that we observe is also in line with previous observations showing that side to side is the most common scanning behavior in *Pipistrellus pipistrellus* (Seibert et al., 2013). However, additional microphones displaced horizontally are needed to completely confirm the side‐to‐side scanning behavior. We do not see a clear indication of the downward scanning observed by Jensen and Miller (1999), but the sparsity of our preliminary data could easily account for that.

UAV‐borne microphone arrays clearly hold great potential for studying bat echolocation in previously unexplored environments such as high altitudes, over open water or just above the forest canopy. Compared to single microphone recordings, microphone array systems additionally provide information on flight patterns through acoustic localization, sonar range through source level measurements, and beam aim/attention through measurements of the emitted beam shape. As such, array recordings provide highly detailed data on how bats utilize echolocation to guide flight and capture prey in the night sky. With our system, we are no longer limited in geographic placement of such arrays and can obtain measurements throughout the entire natural range. The system will undoubtably add substantially to our knowledge of general flight and foraging behavior of bats across their natural habitat and provide insight into the general concepts of echolocation as well as species‐specific adaptations to habitat. Additionally, the system holds great potential for fine‐scale surveys of flight behavior, making it an ideal tool to study spatial distribution of bats. Such information would aid greatly in conservation efforts when planning new infrastructure, for example, surveying flyways around construction sites and around windfarms, where more detailed knowledge of bat flight and echolocation behavior could help mitigate bat fatalities substantially.

## AUTHOR CONTRIBUTIONS

**Christian Jespersen:** Methodology (supporting); writing – review and editing (supporting). **David Docherty:** Methodology (supporting); software (supporting); writing – review and editing (supporting). **John Hallam:** Conceptualization (equal); methodology (equal); software (lead); writing – review and editing (supporting). **Carsten Albertsen:** Methodology (supporting). **Lasse Jakobsen:** Conceptualization (equal); data curation (lead); funding acquisition (lead); methodology (equal); writing – original draft (lead); writing – review and editing (lead).

## FUNDING INFORMATION

The study was funded by DFF grant 8021‐00155A to L.J. and C.J., Villum fonden young investigator grant 00025380 to L.J., and an internal SDU drone grant to L.J., J.H., and D.D.

## CONFLICT OF INTEREST

All authors declare no conflict of interest.

## Supporting information


Figure S1

Table S1

## Data Availability

Data are available via Zendo: Jespersen et al. (2022).
