# Supplementary material for: Drone exploration of bat echolocation: A UAV‐borne multimicrophone array to study bat echolocation
Source: Ecol Evol. 2022 Dec 3;12(12):e9577. doi: 10.1002/ece3.9577 (PMC9719081; doi:10.1002/ece3.9577)

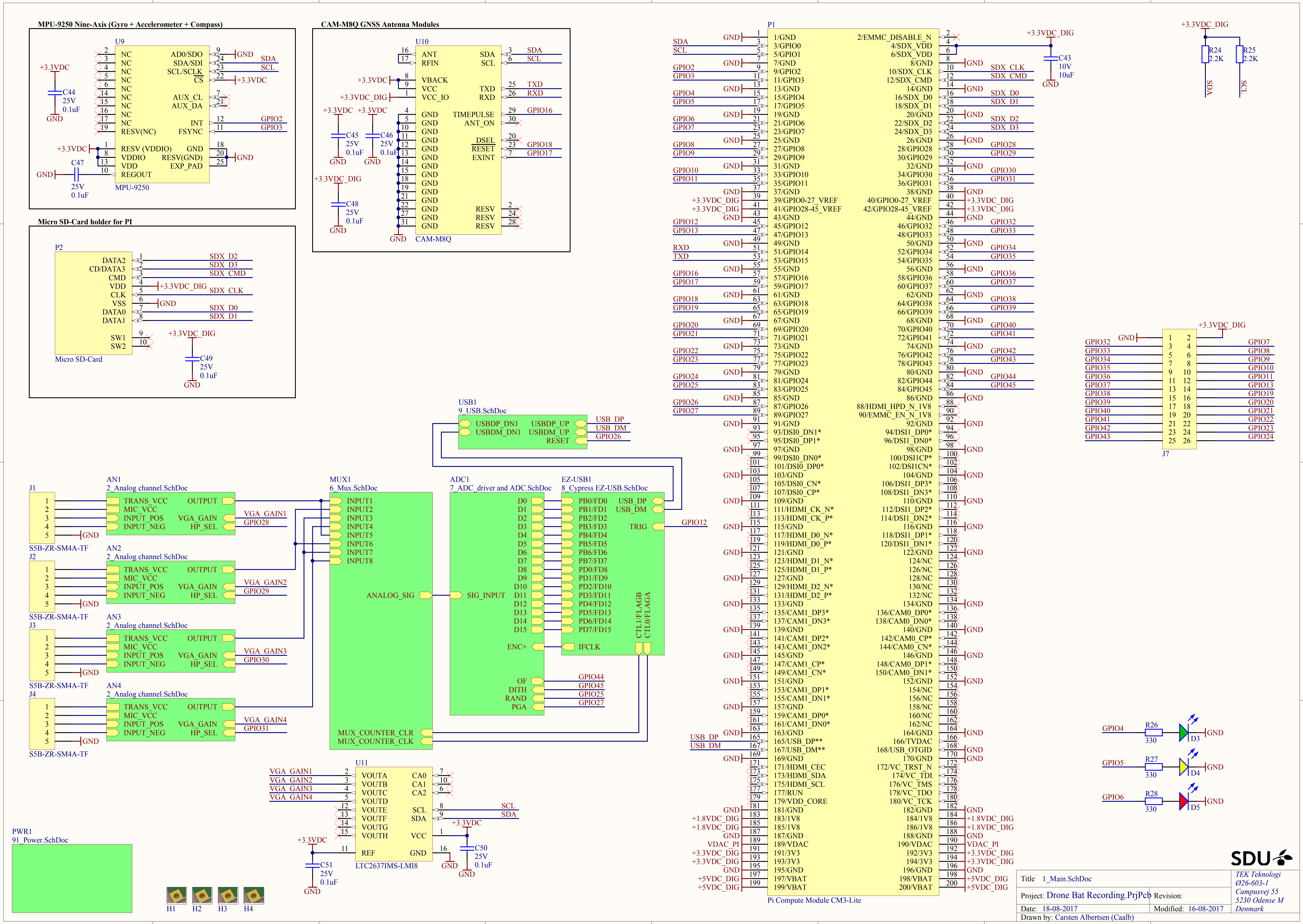
MPU-9250 Nine-Axis (Gyro + Accelerometer + Compass)
U9
MPU-9250
AD0/SDO
SDA/SDI
SCL/SCLK
CS
AUX_CL
AUX_DA
INT
FSYNC
RESV (VDDIO)
VDDIO
VDD
REGOUT
RESV(NC)
RESV(GND)
EXP_PAD
C44 25V 0.1uF
C47 25V 0.1uF
+3.3VDC
GND
SDA
SCL
GPIO2
GPIO3
CAM-M8Q GNSS Antenna Modules
U10
CAM-M8Q
ANT
RFIN
VBACK
VCC
VCC_IO
TXD
RXD
TIMEPULSE
ANT_ON
DSEL
RESET
EXINT
RESV
C45 25V 0.1uF
C46 25V 0.1uF
C48 25V 0.1uF
+3.3VDC_DIG
GPIO16
GPIO18
GPIO17
Micro SD-Card holder for PI
P2
Micro SD-Card
DATA2
CD/DATA3
CMD
VDD
CLK
VSS
DATA0
DATA1
SW1
SW2
SDX_D2
SDX_D3
SDX_CMD
SDX_CLK
SDX_D0
SDX_D1
C49 25V 0.1uF
USB1
9_USB.SchDoc
USBDP_DN1
USBDM_DN1
USBDP_UP
USBDM_UP
RESET
USB_DP
USB_DM
GPIO26
J1
J2
J3
J4
S5B-ZR-SM4A-TF
AN1
AN2
AN3
AN4
2_Analog channel.SchDoc
TRANS_VCC
MIC_VCC
INPUT_POS
INPUT_NEG
OUTPUT
VGA_GAIN
HP_SEL
VGA_GAIN1
VGA_GAIN2
VGA_GAIN3
VGA_GAIN4
GPIO28
GPIO29
GPIO30
GPIO31
MUX1
6_Mux.SchDoc
INPUT1
INPUT2
INPUT3
INPUT4
INPUT5
INPUT6
INPUT7
INPUT8
ANALOG_SIG
MUX_COUNTER_CLR
MUX_COUNTER_CLK
ADC1
7_ADC_driver and ADC.SchDoc
SIG_INPUT
D0 D1 D2 D3 D4 D5 D6 D7 D8 D9 D10 D11 D12 D13 D14 D15
ENC+
OF
DITH
RAND
PGA
GPIO44
GPIO45
GPIO25
GPIO27
EZ-USB1
8_Cypress EZ-USB.SchDoc
PB0/FD0 PB1/FD1 PB2/FD2 PB3/FD3 PB4/FD4 PB5/FD5 PB6/FD6 PB7/FD7
PD0/FD8 PD1/FD9 PD2/FD10 PD3/FD11 PD4/FD12 PD5/FD13 PD6/FD14 PD7/FD15
IFCLK
USB_DP
USB_DM
TRIG
CTL1/FLAGB
CTL0/FLAGA
GPIO12
U11
LTC2637IMS-LMI8
VOUTA VOUTB VOUTC VOUTD VOUTE VOUTF VOUTG VOUTH
CA0 CA1 CA2
SCL
SDA
VCC
REF
GND
C50 25V 0.1uF
C51 25V 0.1uF
PWR1
91_Power.SchDoc
H1 H2 H3 H4
P1
Pi Compute Module CM3-Lite
1/GND 3/GPIO0 5/GPIO1 7/GND 9/GPIO2 11/GPIO3 13/GND 15/GPIO4 17/GPIO5 19/GND 21/GPIO6 23/GPIO7 25/GND 27/GPIO8 29/GPIO9 31/GND 33/GPIO10 35/GPIO11 37/GND 39/GPIO0-27_VREF 41/GPIO28-45_VREF 43/GND 45/GPIO12 47/GPIO13 49/GND 51/GPIO14 53/GPIO15 55/GND 57/GPIO16 59/GPIO17 61/GND 63/GPIO18 65/GPIO19 67/GND 69/GPIO20 71/GPIO21 73/GND 75/GPIO22 77/GPIO23 79/GND 81/GPIO24 83/GPIO25 85/GND 87/GPIO26 89/GPIO27 91/GND 93/DSI0_DN1* 95/DSI0_DP1* 97/GND 99/DSI0_DN0* 101/DSI0_DP0* 103/GND 105/DSI0_CN* 107/DSI0_CP* 109/GND 111/HDMI_CK_N* 113/HDMI_CK_P* 115/GND 117/HDMI_D0_N* 119/HDMI_D0_P* 121/GND 123/HDMI_D1_N* 125/HDMI_D1_P* 127/GND 129/HDMI_D2_N* 131/HDMI_D2_P* 133/GND 135/CAM1_DP3* 137/CAM1_DN3* 139/GND 141/CAM1_DP2* 143/CAM1_DN2* 145/GND 147/CAM1_CP* 149/CAM1_CN* 151/GND 153/CAM1_DP1* 155/CAM1_DN1* 157/GND 159/CAM1_DP0* 161/CAM1_DN0* 163/GND 165/USB_DP** 167/USB_DM** 169/GND 171/HDMI_CEC 173/HDMI_SDA 175/HDMI_SCL 177/RUN 179/VDD_CORE 181/GND 183/1V8 185/1V8 187/GND 189/VDAC 191/3V3 193/3V3 195/GND 197/VBAT 199/VBAT
2/EMMC_DISABLE_N 4/SDX_VDD 6/SDX_VDD 8/GND 10/SDX_CLK 12/SDX_CMD 14/GND 16/SDX_D0 18/SDX_D1 20/GND 22/SDX_D2 24/SDX_D3 26/GND 28/GPIO28 30/GPIO29 32/GND 34/GPIO30 36/GPIO31 38/GND 40/GPIO0-27_VREF 42/GPIO28-45_VREF 44/GND 46/GPIO32 48/GPIO33 50/GND 52/GPIO34 54/GPIO35 56/GND 58/GPIO36 60/GPIO37 62/GND 64/GPIO38 66/GPIO39 68/GND 70/GPIO40 72/GPIO41 74/GND 76/GPIO42 78/GPIO43 80/GND 82/GPIO44 84/GPIO45 86/GND 88/HDMI_HPD_N_1V8 90/EMMC_EN_N_1V8 92/GND 94/DSI1_DP0* 96/DSI1_DN0* 98/GND 100/DSI1CP* 102/DSI1CN* 104/GND 106/DSI1_DP3* 108/DSI1_DN3* 110/GND 112/DSI1_DP2* 114/DSI1_DN2* 116/GND 118/DSI1_DP1* 120/DSI1_DN1* 122/GND 124/NC 126/NC 128/NC 130/NC 132/NC 134/GND 136/CAM0_DP0* 138/CAM0_DN0* 140/GND 142/CAM0_CP* 144/CAM0_CN* 146/GND 148/CAM0_DP1* 150/CAM0_DN1* 152/GND 154/NC 156/NC 158/NC 160/NC 162/NC 164/GND 166/TVDAC 168/USB_OTGID 170/GND 172/VC_TRST_N 174/VC_TDI 176/VC_TMS 178/VC_TDO 180/VC_TCK 182/GND 184/1V8 186/1V8 188/GND 190/VDAC 192/3V3 194/3V3 196/GND 198/VBAT 200/VBAT
+1.8VDC_DIG
VDAC_PI
+5VDC_DIG
RXD
TXD
C43 10V 10uF
R24 2.2K
R25 2.2K
J7
R26 330
R27 330
R28 330
D3
D4
D5
GPIO4
GPIO5
GPIO6
SDU
Title 1_Main.SchDoc
Project: Drone Bat Recording.PrjPcb
Revision:
Date: 18-08-2017
Modified: 16-08-2017
Drawn by: Carsten Albertsen (Caalb)
TEK Teknologi
Ø26-603-1
Campusvej 55
5230 Odense M
Denmark

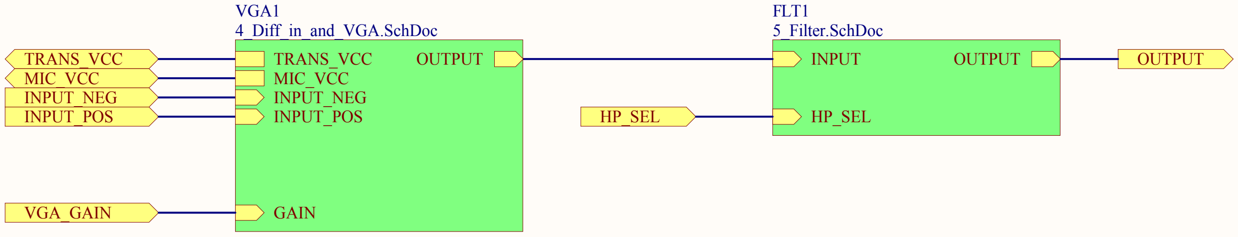

SDU

Title 2_Analog channel.SchDoc		*TEK Teknologi*<br>*Ø26-603-1*<br>*Campusvej 55*<br>*5230 Odense M*<br>*Denmark*
Project: Drone Bat Recording.PrjPcb	Revision:	
Date: 18-08-2017	Modified: 17-08-2017	
Drawn by: Carsten Albertsen (Caalb)		

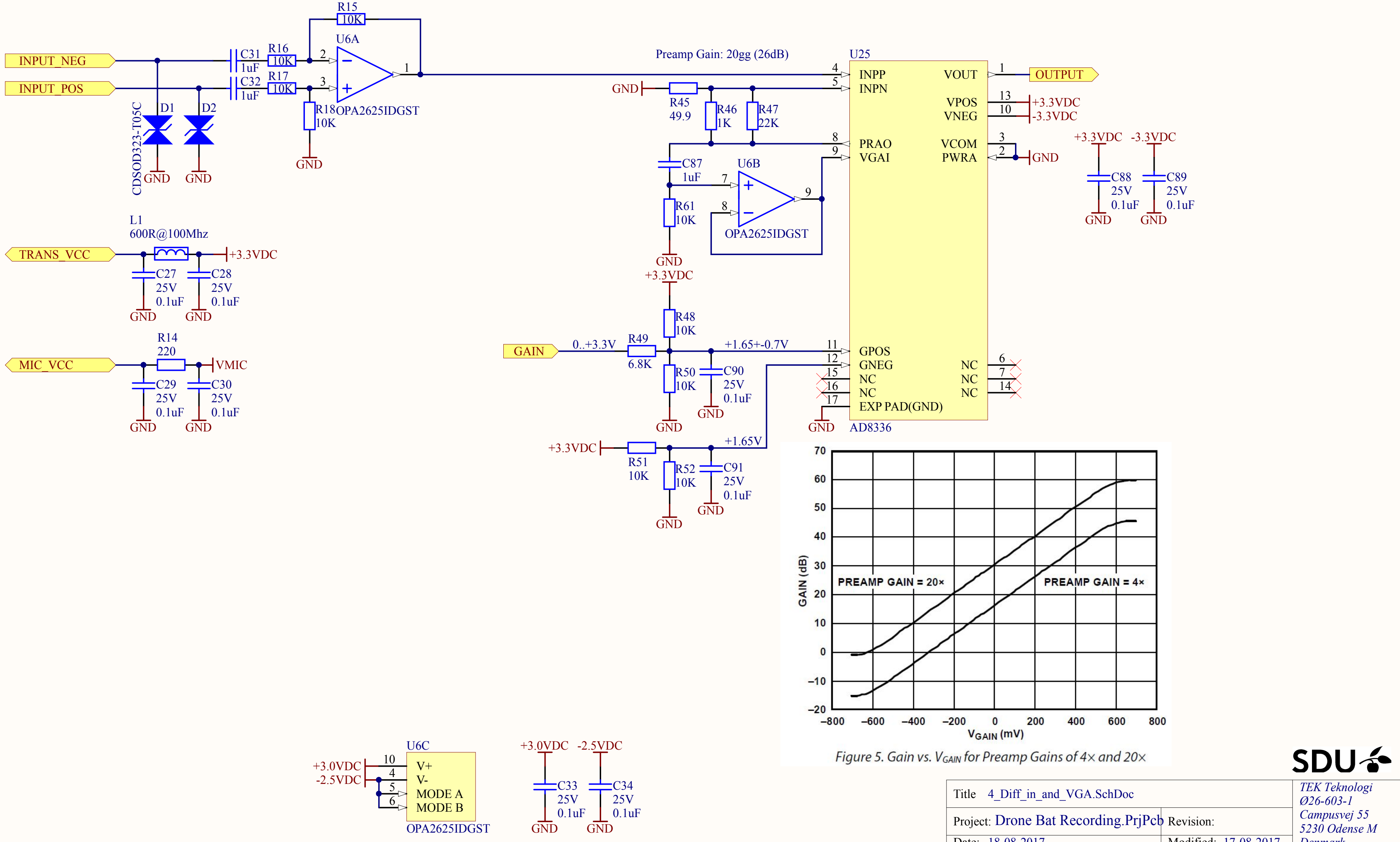

Figure 5. Gain vs. V_{GAIN} for Preamp Gains of 4× and 20×

Title	4_Diff_in_and_VGA.SchDoc
Project:	Drone Bat Recording.PrjPcb
Revision:	
Date:	18-08-2017
Modified:	17-08-2017
Drawn by:	Carsten Albertsen (Caalb)

TEK Teknologi
Ø26-603-1
Campusvej 55
5230 Odense M
Denmark

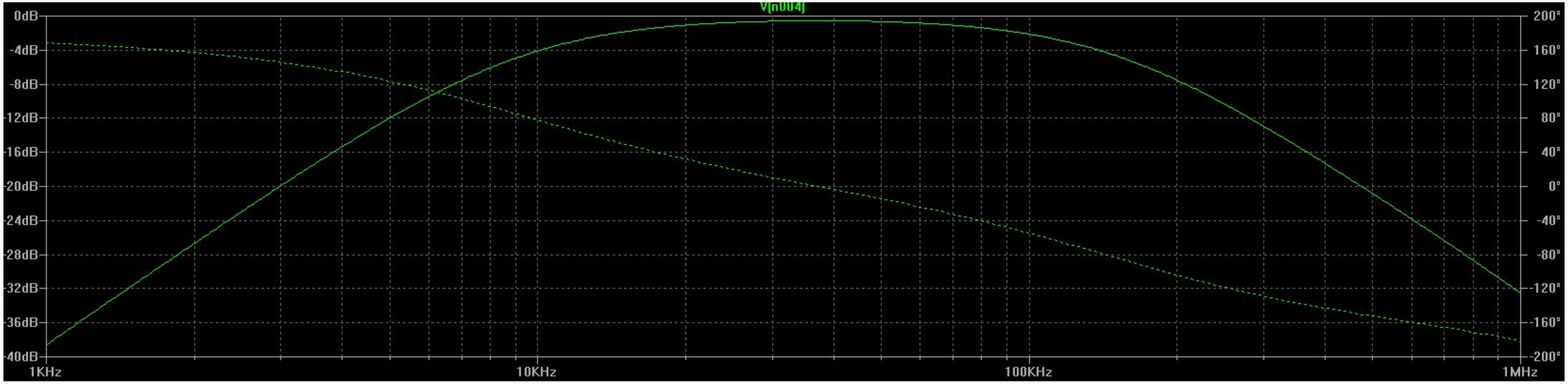

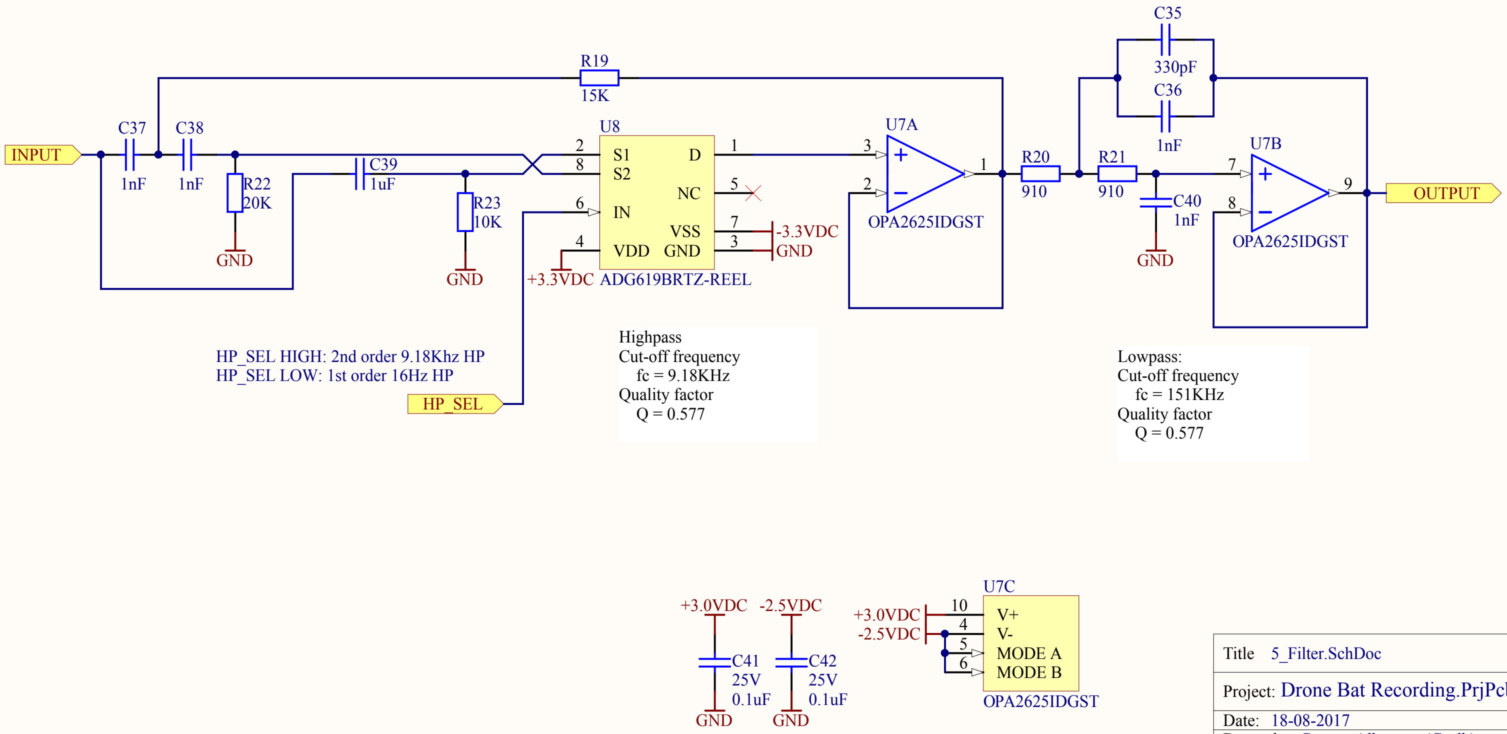

SDU

Title 5_Filter.SchDoc		*TEK Teknologi Ø26-603-1 Campusvej 55 5230 Odense M Denmark*
Project: Drone Bat Recording.PrjPcb	Revision:	
Date: 18-08-2017	Modified: 16-08-2017	
Drawn by: Carsten Albertsen (Caalb)		

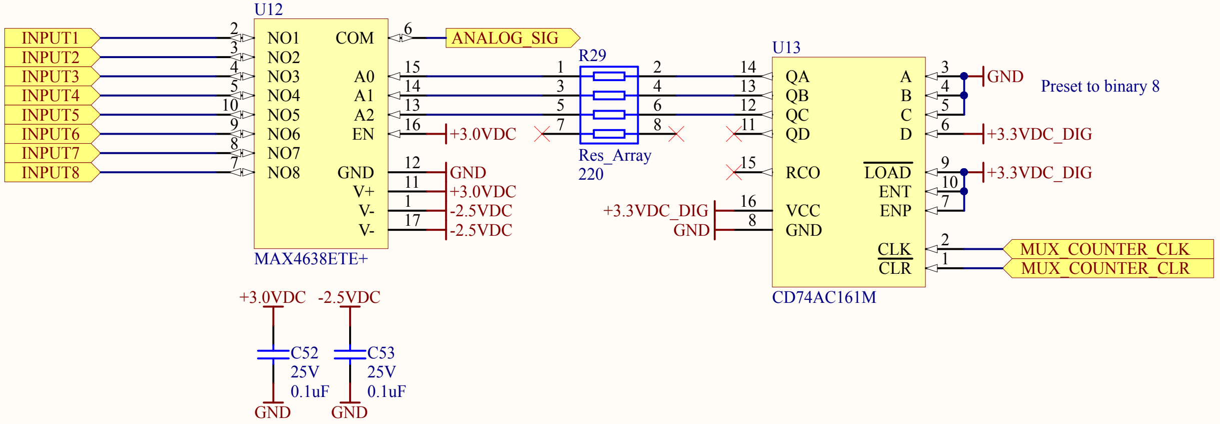

Title 6_Mux.SchDoc	
Project: Drone Bat Recording.PrjPcb	Revision:
Date: 18-08-2017	Modified: 18-08-2017
Drawn by: Carsten Albertsen (Caalb)	

TEK Teknologi
Ø26-603-1
Campusvej 55
5230 Odense M
Denmark

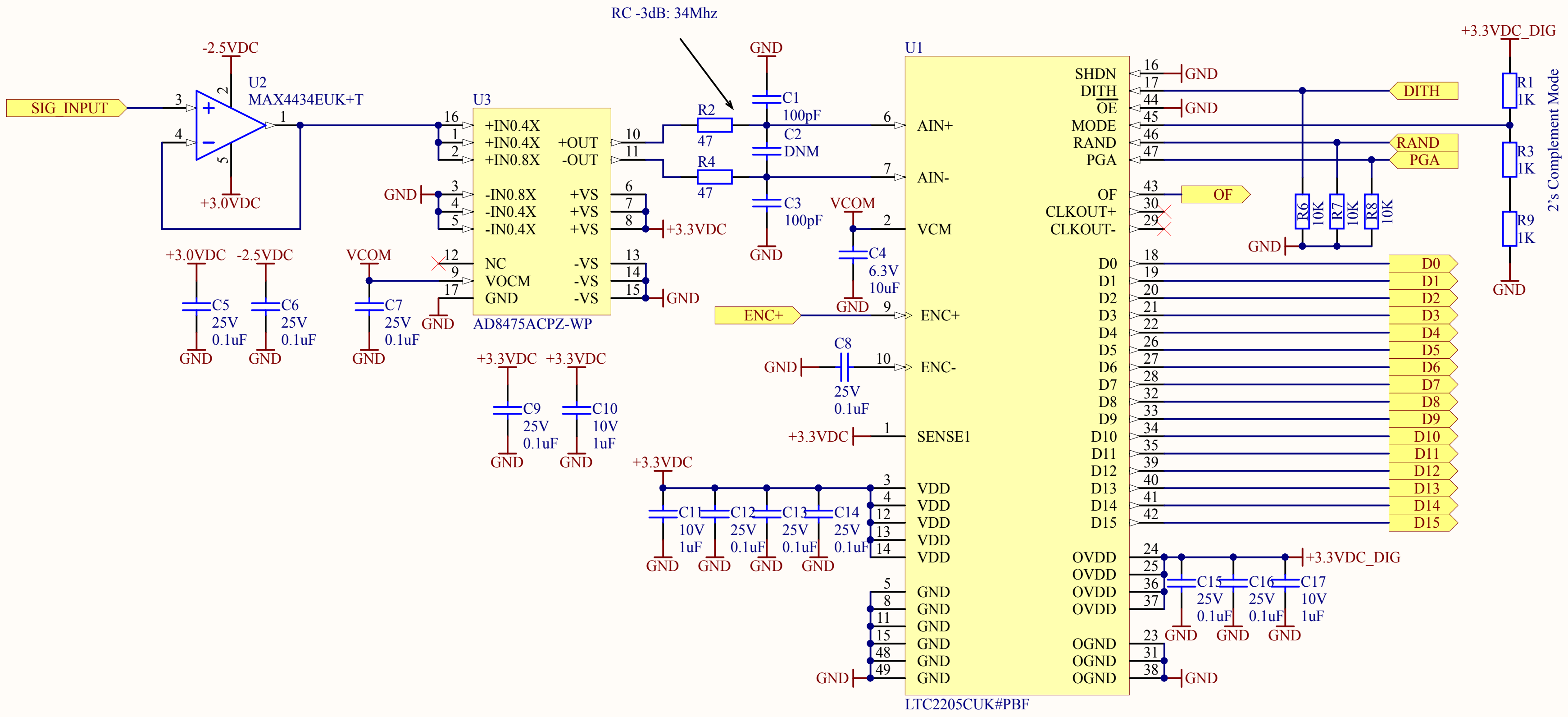

SDU

Title 7_ADC_driver and ADC.SchDoc

Project: Drone Bat Recording.PrjPcb Revision:

Date: 18-08-2017 Modified: 16-08-2017

Drawn by: Carsten Albertsen (Caalb)

TEK Teknologi
Ø26-603-1
Campusvej 55
5230 Odense M
Denmark

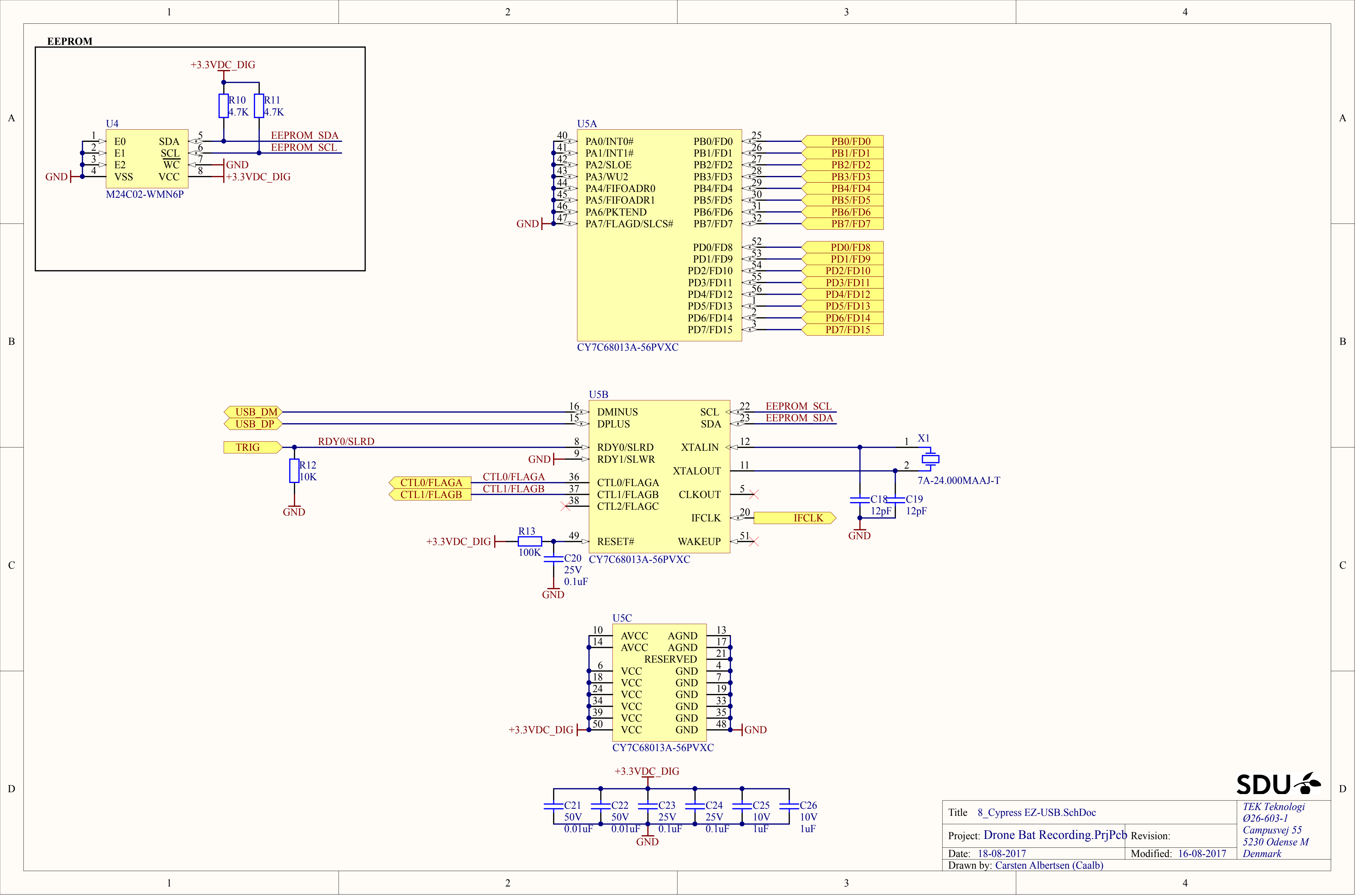
1 2 3 4
A B C D
EEPROM
+3.3VDC_DIG
R10 4.7K
R11 4.7K
U4
E0 SDA
E1 SCL
E2 WC
VSS VCC
1 2 3 4 5 6 7 8
EEPROM_SDA
EEPROM_SCL
GND
+3.3VDC_DIG
GND
M24C02-WMN6P
U5A
PA0/INT0# PA1/INT1# PA2/SLOE PA3/WU2 PA4/FIFOADR0 PA5/FIFOADR1 PA6/PKTEND PA7/FLAGD/SLCS#
40 41 42 43 44 45 46 47
GND
PB0/FD0 PB1/FD1 PB2/FD2 PB3/FD3 PB4/FD4 PB5/FD5 PB6/FD6 PB7/FD7
25 26 27 28 29 30 31 32
PD0/FD8 PD1/FD9 PD2/FD10 PD3/FD11 PD4/FD12 PD5/FD13 PD6/FD14 PD7/FD15
52 53 54 55 56 1 2 3
CY7C68013A-56PVXC
U5B
USB_DM
USB_DP
TRIG
RDY0/SLRD
R12 10K
GND
CTL0/FLAGA
CTL1/FLAGB
16 DMINUS
15 DPLUS
8 RDY0/SLRD
9 RDY1/SLWR
36 CTL0/FLAGA
37 CTL1/FLAGB
38 CTL2/FLAGC
49 RESET#
SCL 22 EEPROM_SCL
SDA 23 EEPROM_SDA
XTALIN 12
XTALOUT 11
CLKOUT 5
IFCLK 20 IFCLK
WAKEUP 51
X1
1 2
7A-24.000MAAJ-T
C18 12pF
C19 12pF
GND
+3.3VDC_DIG
R13 100K
C20 25V 0.1uF
GND
CY7C68013A-56PVXC
U5C
10 AVCC
14 AVCC
6 VCC
18 VCC
24 VCC
34 VCC
39 VCC
50 VCC
AGND 13
AGND 17
RESERVED 21
GND 4
GND 7
GND 19
GND 33
GND 35
GND 48
+3.3VDC_DIG
GND
CY7C68013A-56PVXC
+3.3VDC_DIG
C21 50V 0.01uF
C22 50V 0.01uF
C23 25V 0.1uF
C24 25V 0.1uF
C25 10V 1uF
C26 10V 1uF
GND
SDU
Title 8_Cypress EZ-USB.SchDoc
Project: Drone Bat Recording.PrjPcb
Revision:
Date: 18-08-2017
Modified: 16-08-2017
Drawn by: Carsten Albertsen (Caalb)
TEK Teknologi
Ø26-603-1
Campusvej 55
5230 Odense M
Denmark

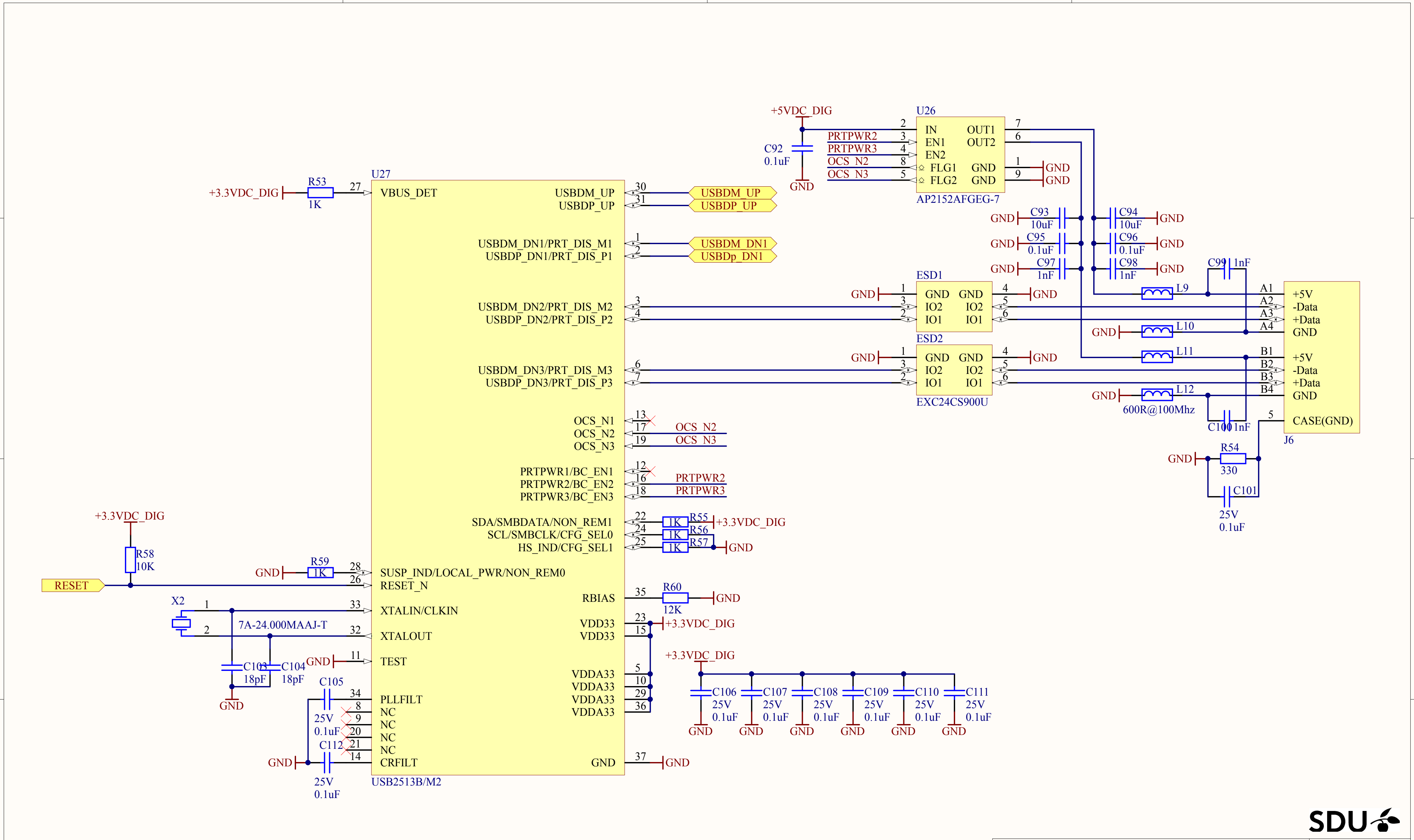

Title 9_USB.SchDoc		*TEK Teknologi*
Project: Drone Bat Recording.PrjPcb	Revision:	*Ø26-603-1* *Campusvej 55* *5230 Odense M*
Date: 18-08-2017	Modified: 15-08-2017	*Denmark*
Drawn by: Carsten Albertsen (Caalb)		

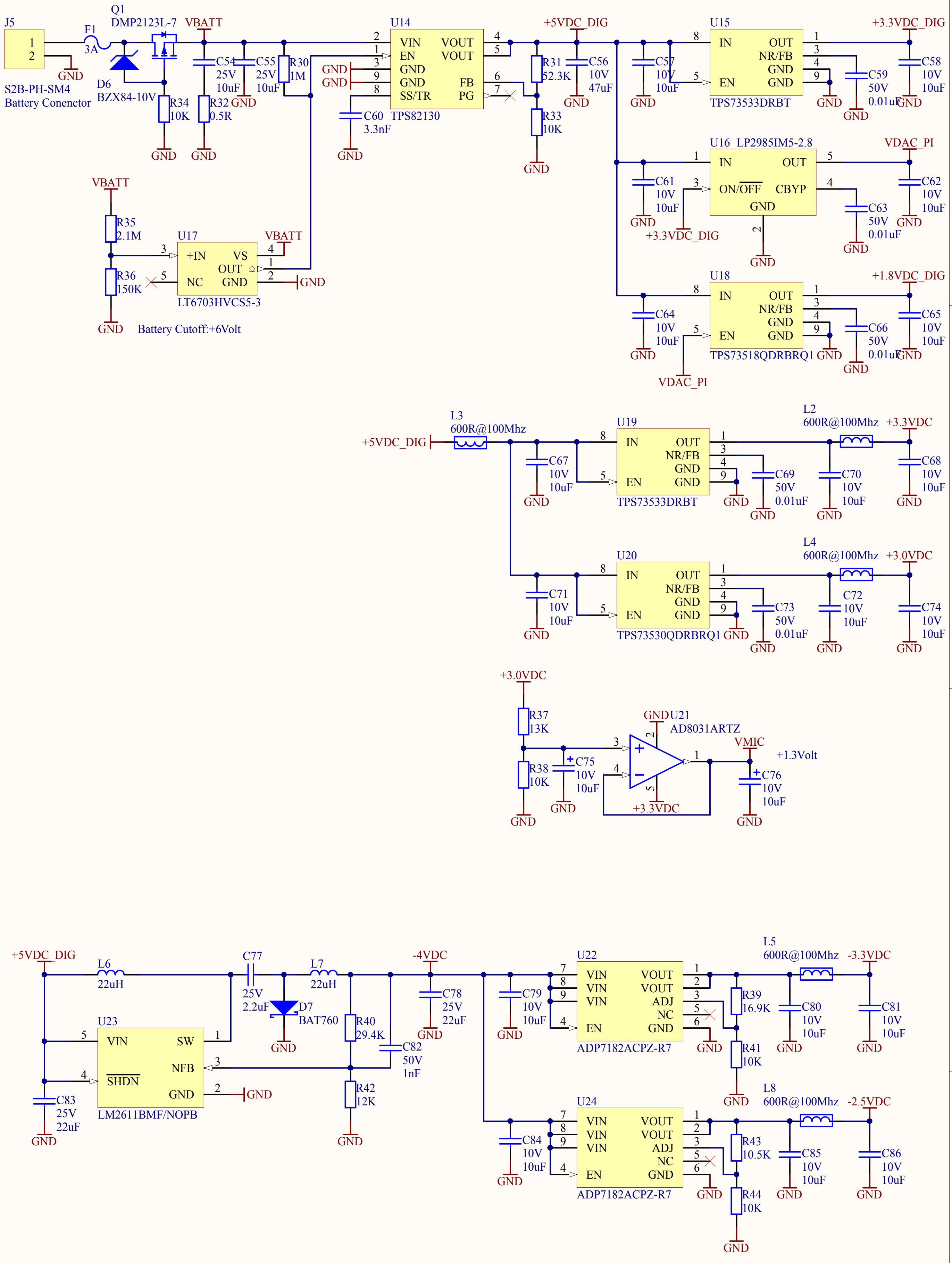

SDU

Title 91_Power.SchDoc		*TEK Teknologi*
Project: Drone Bat Recording.PrjPcb	Revision:	*Ø26-603-1* *Campusvej 55* *5230 Odense M*
Date: 18-08-2017	Modified: 17-08-2017	*Denmark*
Drawn by: Carsten Albertsen (Caalb)		

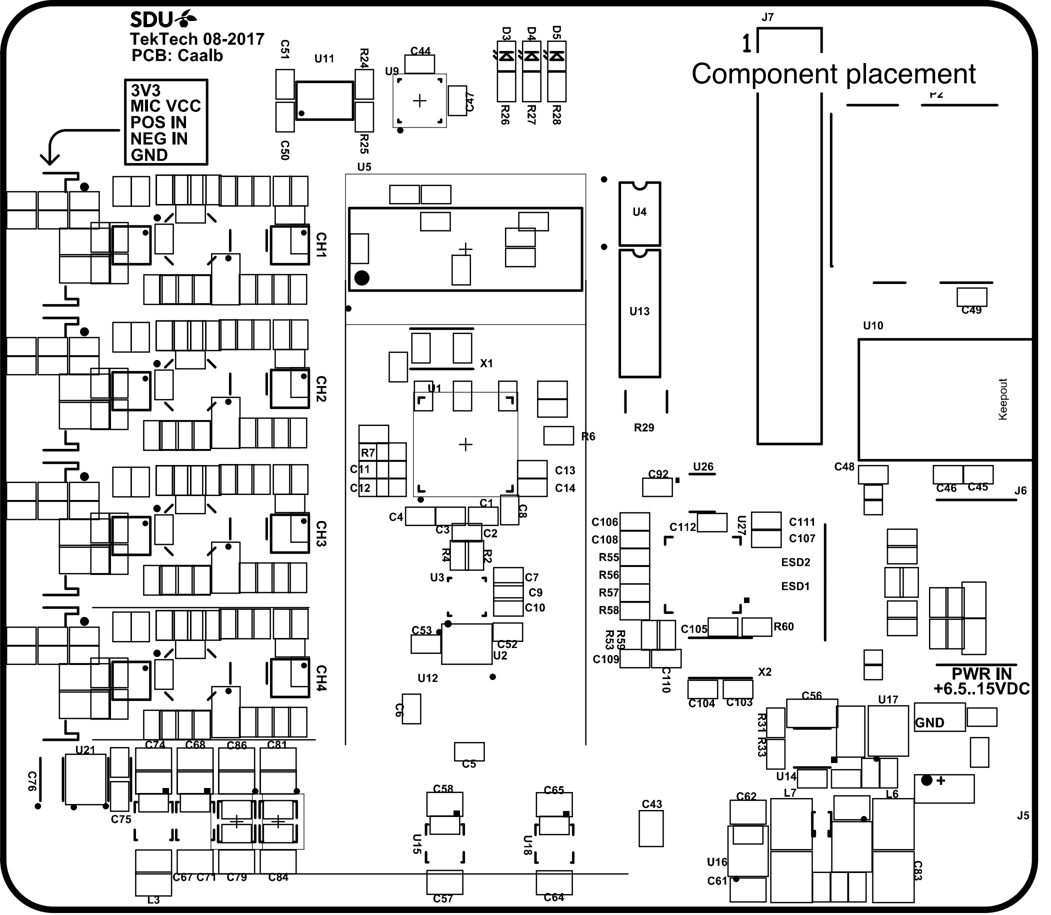
SDU
TekTech 08-2017
PCB: Caalb
3V3
MIC VCC
POS IN
NEG IN
GND
Component placement
J7
1
P2
C51
U11
R24
C50
R25
C44
U9
C47
D3
D4
D5
R26
R27
R28
U5
U4
U13
CH1
CH2
CH3
CH4
X1
U1
R7
C11
C12
R6
C13
C14
R29
U10
Keepout
C49
C48
C46
C45
J6
C92
U26
C4
C1
C8
C3
C2
R4
R2
U3
C7
C9
C10
C53
C52
U2
U12
C6
C5
C106
C108
R55
R56
R57
R58
C112
U27
C111
C107
ESD2
ESD1
C105
R60
R53
R59
C109
C110
X2
C104
C103
PWR IN
+6.5..15VDC
C56
U17
GND
R31
R33
U14
L7
L6
C62
U16
C61
C83
J5
C43
U21
C76
C74
C68
C86
C81
C75
C67
C71
C79
C84
L3
C58
U15
C57
C65
U18
C64

PCB top side 3D view

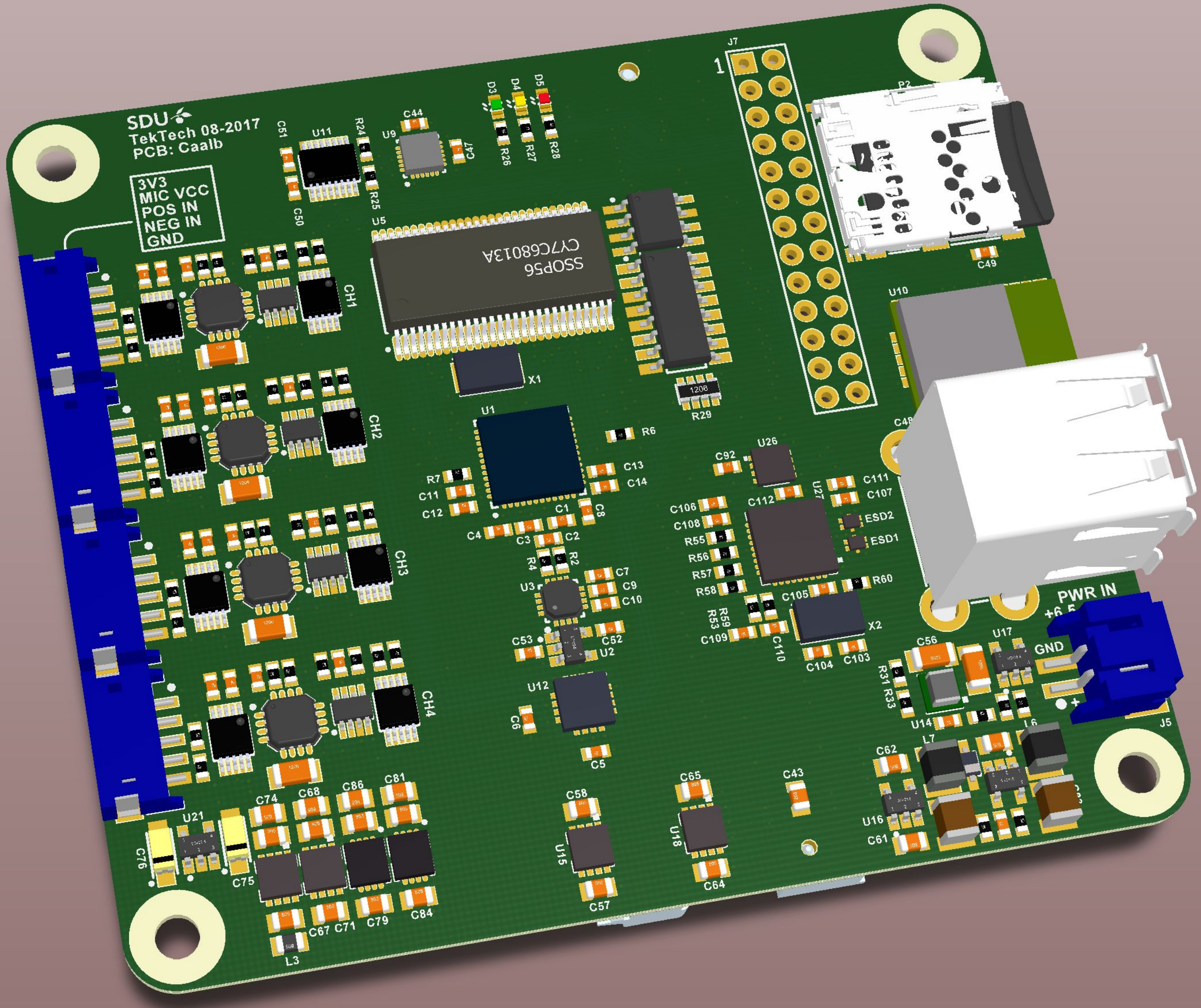

PCB bottom side 3D view

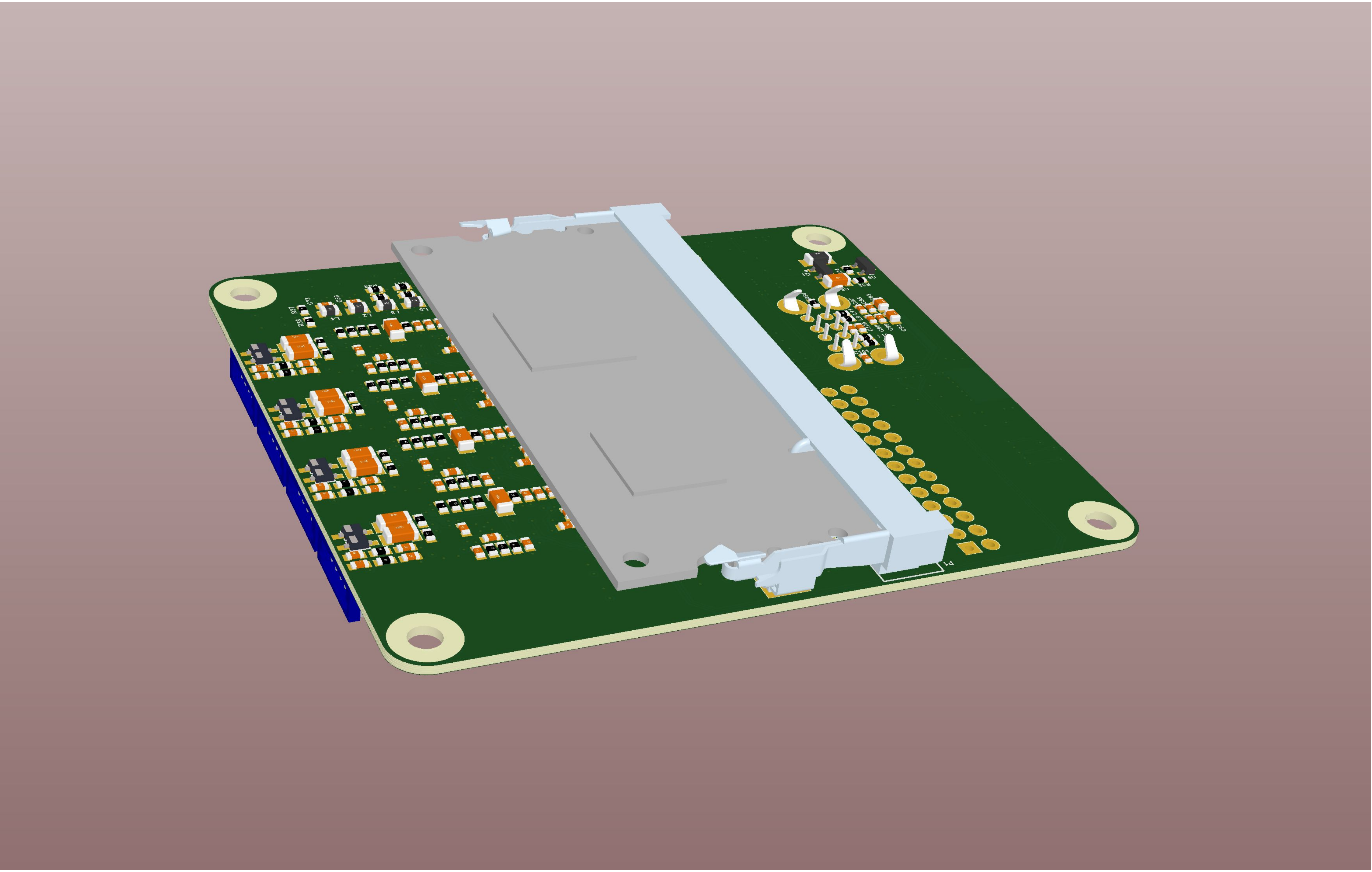

Supplement: Supplementary file 1 — Figure S1 [file ECE3-12-e9577-s001.pdf]
