# Supplementary figures and images for: Drone exploration of bat echolocation: A UAV‐borne multimicrophone array to study bat echolocation
Source: Ecol Evol. 2022 Dec 3;12(12):e9577. doi: 10.1002/ece3.9577 (PMC9719081; doi:10.1002/ece3.9577)

Address:  
TEK Teknologi  
6226-663-1  
Campuswaj SS  
6226 Odenas M

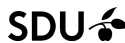

Supplement: Supplementary file 2 — Table S1 [file ECE3-12-e9577-s002.pdf]
